# Gestational Weight Gain Counseling Insights from Healthcare Providers and Saudi Women: Riyadh Mother and Baby Follow-Up Study (RAHMA Explore)

**DOI:** 10.3390/healthcare14030403

**Published:** 2026-02-05

**Authors:** Amel Fayed, Samia Esmaeil, Alya Khalid AlZabin, Wijdan Awad Almutiri, Ebtesam Hoshan Almajed, Hayfaa Wahabi

**Affiliations:** 1 Department of Family and Community Medicine, College of Medicine, Princess Nourah bint Abdulrahman University, P.O. Box 84428, Riyadh 11671, Saudi Arabia; 2 Research Chair for Evidence-Based Health Care and Knowledge Translation, King Saud University, P.O. Box 7805, Riyadh 11451, Saudi Arabiahwahabi@ksu.edu.sa (H.W.); 3 Department of Family and Community Medicine, College of Medicine, King Saud University, P.O. Box 7805, Riyadh 11451, Saudi Arabia; 4 College of Medicine, Princess Nourah bint Abdulrahman University, P.O. Box 84428, Riyadh 11671, Saudi Arabia

**Keywords:** gestational weight gain target, healthcare provider counseling, prepregnancy weight

## Abstract

**Background**: Monitoring and managing gestational weight gain (GWG) during antenatal care (ANC) is linked to better maternal and neonatal outcomes. The Institute of Medicine (IOM) guidelines are based on pre-pregnancy BMI and reduce obstetric risks. Pregnant women’s views and healthcare providers’ (HCPs) practices are key to effective GWG counseling. This study aims to: (1) investigate the proportion of women who received GWG advice per IOM guidelines, and (2) explore HCP practices and views on GWG counseling. **Methods**: This is a cross-sectional study of Saudi pregnant women who delivered within one year of the study and HCPs who provided ANC. Women provided data on demographics, pre-pregnancy BMI, recall of GWG advice, and their target GWG. HCPs rated their knowledge and counseling practices. **Results:** Of 1151 women, 48.8% were pre-pregnancy overweight or obese, 47.6% were normal weight, and 3.6% were underweight. Most women (74.5%) received no GWG advice, and only 8.8% followed IOM guidelines. Women with obesity and overweight were more likely to receive correct advice (15.5% and 11.5%), compared to 5.3% of normal-weight and 2.4% underweight women. Overweight and obese women were more likely to define the correct GWG (AOR = 2.84 and 5.85). Receiving proper advice greatly increased the likelihood of proper GWG definition (AOR = 7.13). Among 28 HCPs, 53.6% reported that women rarely ask about the GWG target. Nearly 93% of them weigh women at each visit, but only 21.4% set personalized GWG targets. Most HCPs (82.2%) viewed discussing GWG as a high priority, and 70% felt confident providing guidance on GWG, diet, and exercise. **Conclusions:** Many women receive no GWG guidance, and most advice does not align with IOM guidelines. Enhancing Saudi women’s knowledge regarding GWG targets through health education, in conjunction with ongoing medical education for healthcare professionals concerning guidelines for GWG, represents modifiable factors and a critical opportunity to foster healthier pregnancy outcomes.

## 1. Introduction

Adequate gestational weight gain (GWG) is consistently linked to better maternal and neonatal outcomes, whereas inadequate and excessive GWG were associated with preterm delivery, cesarean section, low birth weight, and macrosomia [1]. The effect may extend to early childhood obesity, metabolic morbidities, especially increased risks of type 1 diabetes and other metabolic disorders among children of women with high pre-pregnancy Body Mass Index (BMI) and higher GWG, making it crucial to incorporate these findings when managing BMI both pre-pregnancy and during pregnancy [2,3].

Monitoring GWG during antenatal care (ANC) visits is one of the essential recommendations of the World Health Organization (WHO) [4] and the American Association of Obstetrics and Gynecology which recommends that Healthcare Providers (HCPs) should calculate the Body Mass Index (BMI) of women during their initial ANC visits and set their target GWG according to Institute of Medicine (IOM) standards [5].

GWG monitoring serves not only as a screening tool for early adverse outcomes such as gestational diabetes or hypertension but also as an effective strategy for promoting healthier weight control through counselling and patients’ involvement. Furthermore, strong evidence demonstrates that structured nutritional and physical activity programs provided during pregnancy successfully mitigated excessive GWG and supported women in achieving their target GWG [6,7,8].

Despite this guidance, earlier studies reported a wide range of receipt of GWG advice among pregnant women, from 26% to 81%. Moreover, only 12–48% reported receiving the advice in accordance with the IOM guidelines [9]. Additionally, a recent systematic review of GWG among more than one million women found that only 21–36% of women achieved adequate GWG, and among obese and overweight women, nearly half of the women exceeded the target GWG (60–64%). In contrast, 43% of underweight women failed to achieve the target GWG [1,10].

Both women’s perspectives and physicians’ views are crucial to effective GWG counseling. Understanding women’s beliefs, preferences, and concerns about pregnancy weight gain helps ensure that counseling is personalized and responsive to their needs. At the same time, physicians provide clinical expertise and evidence-based guidance that can help prevent complications associated with inadequate or excessive GWG. When both viewpoints are considered, communication becomes more collaborative, fostering trust and improving adherence to recommendations. This shared approach supports healthier outcomes for both mother and baby, and promotes a more positive and informed pregnancy experience [11].

The Riyadh Mother and Baby (RAHMA) multicenter study is the largest cohort study investigating pregnancy outcomes among Saudi women, comprising comprehensive data on 14,568 mother–child pairs. One of the main findings of the RAHMA study was that inadequate GWG was reported in nearly 42% of Saudi pregnant women, excessive GWG in 26%, and adequate GWG in only 32%. Additionally, the study highlighted the importance of GWG and examined the independent effects of GWG and pre-pregnancy obesity on pregnancy outcomes among Saudi women. It confirmed that excessive GWG increases the risk of pregnancy-related hypertension and emphasized that pre-pregnancy obesity increases the risk of gestational diabetes, hypertensive disorders during pregnancy [12], and emergency cesarean section.

In Saudi Arabia, few studies [13,14] have examined pregnant women’s awareness of GWG and their perceptions of counseling and maternal nutrition. However, we found no published study that directly examines physicians’ views or counseling practices regarding GWG in this context.

As part of the ongoing RAHMA (explore) research series, the objectives of this study are:

1. To estimate the proportion of women who received GWG advice during pregnancy and determine its accuracy according to the IOM.
2. To explore the viewpoints and professional practices of HCPs concerning GWG counseling.

## 2. Methods

### 2.1. Study Design, Settings, and Participants

The research employed a cross-sectional design targeting two separate populations: maternal participants (women currently pregnant or within the first year postpartum) and the healthcare professionals who provide care to pregnant women. The participating women were recruited by sharing the invitation link on social media platforms using a snowballing non-probability sampling technique. Women were eligible to participate in the study if they were Saudi, currently pregnant, or had given birth to at least one baby within the last year, and if they agreed to participate.

HCPs were recruited using a purposive non-probability sampling method from primary healthcare centers or hospitals providing ANC. To be eligible, HCPs must meet two criteria: routine involvement in ANC for at least one year and membership in one of these professional groups: obstetricians/gynecologists, family medicine physicians, general practitioners, registered nurses, or dietitians.

The invitation links included a cover page that clarified the study’s objectives, and electronic informed consent was obtained in advance. Participation was voluntary and anonymous; both surveys took approximately 5–8 min to complete, and all incomplete questionnaires were automatically deleted from the database before data management and analysis. 

### 2.2. Sample Size Calculation

For the group of women in the current study, assuming that at least 10% [15] (±5%) would recall receiving medical advice about GWG, with a power of 99% (beta = 0.01) and a confidence level of 95% (alpha = 0.05), the minimum sample size required was 912. For healthcare providers, assuming 80 to 95% monitor the GWG of their patients during ANC visits, with a power of 80% (beta = 0.2) and a confidence level of 95% (alpha = 0.05), the minimum sample size required was 28.

Despite using non-probability sampling techniques that might have introduced selective bias or underrepresentation of some groups, the large sample of women enabled us to recruit a large number of women with various experiences of pregnancy (currently pregnant or new mothers) across all BMI categories. Additionally, to ensure data quality, we used validated Arabic-language data collection tools for the women and restricted participation to one response per device.

### 2.3. Data Collection Method

We collected and managed the data using RedCap, a secure web-based platform for research data capture hosted at Princess Nourah Bint Abdulrahman University. To prevent duplicate entries, responses were limited to one per device. The questionnaire cover page provided a brief description of the study objectives and assured the confidentiality of the data and the voluntary nature of participation. An electronic consent form was included, with an option to agree or decline to participate. Upon selecting the agree button, the full survey was loaded. If participants chose not to agree, a thank-you message was displayed, followed by closure of the survey.

Given the snowballing non-probability technique, it was not possible to determine the invitation or response rates; however, the completion rate was 84% among women and 70% among HCPs.

### 2.4. Study Outcomes and Variables

Development and validation of data collection tools were conducted through a meticulous multi-phase process beginning with a comprehensive literature review. Following the construction of the questionnaire, face and content validity were evaluated by a panel of three experts (two obstetricians and one research methodologist). The panel reviewed and revised the questions, providing independent ratings for appropriateness and relevance to the study objectives. Items with low scores were subsequently removed, while those demonstrating high relevance were retained in the final version. The questionnaire underwent forward and backward translations to ensure linguistic and cultural appropriateness for the Saudi population, which was further assessed through piloting among Saudi women, following evaluation by Saudi members of the research team. This validation process encompassed evaluating face and content validity, as well as verifying the translation process through expert review and piloting the latest version [16,17]. The HCPs’ data collection tool was retained in its English form.

1. **The survey of women:**

The questionnaire addressed the following: (a) Sociodemographic characteristics, including age, education, occupation, latest pre-pregnancy weight, height, and the timing of the last pregnancy. Before conducting the formal analysis, participants with missing weight or height were excluded, as all participants were classified and compared by BMI. (b) Whether participants sought GWG information during the most recent pregnancy. (c) Recall of health provider’s advice on GWG. (d) Women’s definition of the appropriate GWG according to their weight.

We evaluated patients seeking GWG information using one question: “During this pregnancy, have you looked for information (including on the internet) or asked anyone about how much weight you should gain during pregnancy?” {yes/no}. We assessed recall of health provider’s advice on GWG with two questions: (1) Has your healthcare provider given you a specific weight gain suggestion or target for this pregnancy? {yes/no}, and (2) What weight range did the healthcare provider advise you to reach? {nonspecific & four IOM categories}. These questions were adopted from previous literature [18] that validated them. Additionally, we asked about any referrals to dietitians or advice on following specific diets.

We asked participants to identify the appropriate GWG range for them, offering four options according to IOM guidelines. We evaluated the accuracy and the correctness of GWG advice and expectation against IOM guidelines and recorded them as correct or incorrect, considering each woman’s pre-pregnancy BMI.

The Arabic version of the questionnaire is available as Supplementary File S1.

2. **The healthcare providers’ survey:**

The survey gathered information from HCPs about their knowledge, attitudes, and practices regarding GWG, nutrition, and physical activity [19]. They reported the proportion of pregnant patients (on a 1–5 scale, 1: <10% to 5: >90%) for whom they implemented GWG counseling in accordance with IOM guidelines.

Additionally, they rated their knowledge of GWG, nutrition, and physical activity counseling for pregnant women, as well as the appropriateness and availability of referral policies in their hospitals, on a 5-point Likert scale (1: strongly disagree; 5: strongly agree). Lastly, they were asked to indicate their perceived priority for discussing and assessing GWG on a 5-point Likert scale as well.

### 2.5. Statistical Analysis

We used SPSS version 21 (IBM Corp., Released 2012. IBM SPSS Statistics for Windows, Version 21.0. IBM Corp: Armonk, NY, USA) to analyze the data. Descriptive analysis was used to describe the characteristics of the data using frequency and percentage for categorical data, and the Chi-square test or Fisher’s exact test was used for testing the association of categorical variables. A logistic regression model was developed to identify factors associated with proper GWG definition among participating women, as defined by IOM and pre-pregnancy BMI. The factors tested included: age, education, occupation, economic status, pre-pregnancy BMI, pregnancy status, seeking information about GWG, and receiving medical guidance on a GWG target from a health professional during pregnancy. A sensitivity analysis was conducted among pregnant women only to test these factors and to explore the robustness of predictors among women with the least expected recall bias.

### 2.6. Ethical Consideration

This study was conducted in accordance with the Helsinki declaration after Institutional Review Board approval (IRB log number 23-0203, 12 April 2023). Electronic Informed consent was assured by all participants before joining the study.

## 3. Results

This study included 1151 women (after excluding 47 women with missing weight or height) and 28 HCPs who completed the survey. Table 1 summarizes their sociodemographic and clinical characteristics. The mean age of participating women was 36.09 ± 9.04 years, and most held a university degree (71.3%). Nearly half were employed (47.5%), and the majority reported sufficient family income (85.19%). Regarding pregnancy status, 84.62% had been pregnant within the past year, whereas 15.38% were currently pregnant. With respect to pre-pregnancy BMI, 47.6% had a normal BMI, 33.1% were overweight, and 15.7% were obese; only 3.6% were underweight. Among HCPs, more than half specialized in obstetrics and gynecology (53.6%), and 10.7% were nurses. Most providers worked in primary healthcare centers (75%), whereas the remaining 25% were based in hospitals.

Table 2 summarizes the patterns of seeking medical advice about GWG among women by pre-pregnancy BMI. Most women (74.5%) received non-specific medical advice from their HCPs about GWG targets, and only 101 women (8.8%) received advice consistent with IOM guidelines. Women with obesity and overweight were more likely to receive correct advice (15.5% and 11.5%, respectively) than those with normal BMI (5.3%) or underweight (2.4%) (Table 2).

**Figure 1:**
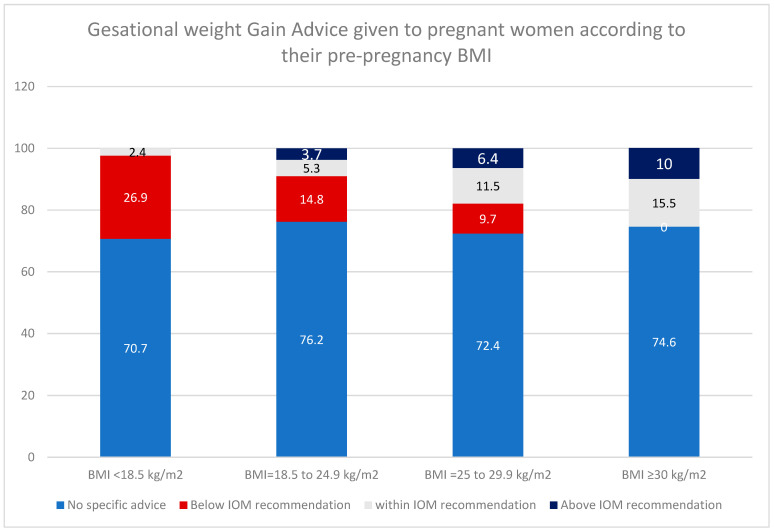
Gestational Weight Gain advice provided to women according to their pre-pregnancy Body Mass Index (BMI).

Furthermore, GWG below IOM recommendations was primarily reported among those with underweight (26.9%) and normal BMI (14.8%), while GWG above IOM recommendations was mainly reported among those with overweight (6.4%) and obesity (10%) (Figure 1).

About one third of overweight and obese women (27.2% and 30.9%, respectively) were advised to follow a specific diet, a rate significantly higher than in the normal/underweight groups. Referral to dietitians was recalled in less than 20% of participants across all groups (*p*-value = 0.86).

Although a similar proportion of women in all groups (more than half) reported searching for or asking about GWG during pregnancy, only 27.9% correctly defined the appropriate GWG target for their BMI. Obese and overweight women were more likely to correctly identify the GWG ranges appropriate for their BMI (54.7% and 35.2%, respectively) than underweight women (9.8%) and those with normal BMI (15.7%; *p* < 0.01).

After adjusting for covariates, pre-pregnancy BMI, higher economic level, and HCP-provided correct advice were the only significant predictors of defining appropriate GWG. Compared with women with normal pre-pregnancy BMI, those with overweight and obesity were two to five times more likely to define the correct GWG (AOR = 2.84, 95% CI 2.01–4.03 and AOR = 5.85, 95% CI 3.88–8.82, respectively). Additionally, correct advice from HCPs significantly increased the likelihood of defining appropriate GWG by about sevenfold compared with those who received non-specific GWG advice (AOR = 7.13, 95% CI 4.35–11.66) (Table 3).

To assess the robustness of the regression model’s findings, a sensitivity analysis was conducted to examine how the same covariates affected the correct definition of appropriate GWG among pregnant women (N = 177, missing =6). The effect of pre-pregnancy BMI was not statistically significant, nor was the positive effect of HCPs’ correct advice. However, the positive effect on the correct definition of appropriate GWG among those with a higher economic level remained statistically significant. Additionally, not seeking information about GWG and receiving incorrect advice from HCPs decreased the likelihood of correctly defining GWG.

**HCPs survey:** In this study, 28 HCPs were included. Of these, 53.6% were obstetricians, 32.1% were general practitioners or family medicine physicians, 10.7% were nurses, and only one dietitian participated. Most worked in primary health care centers, and 25% were based in hospitals (Table 1).

**Figure 2:**
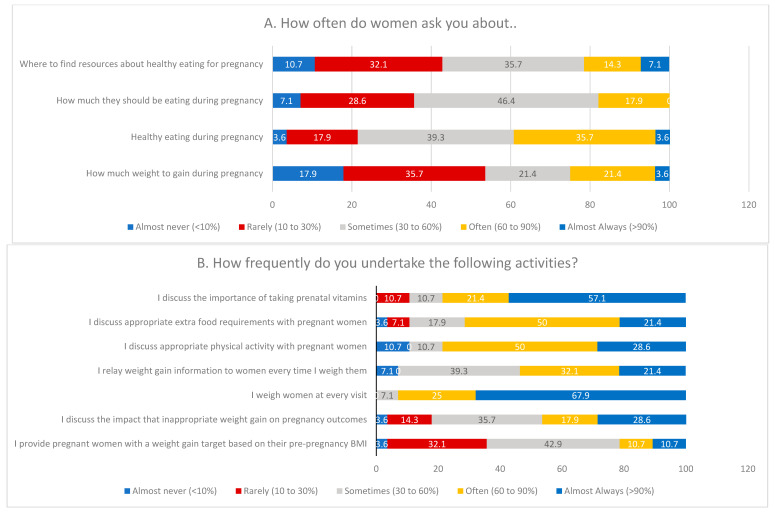

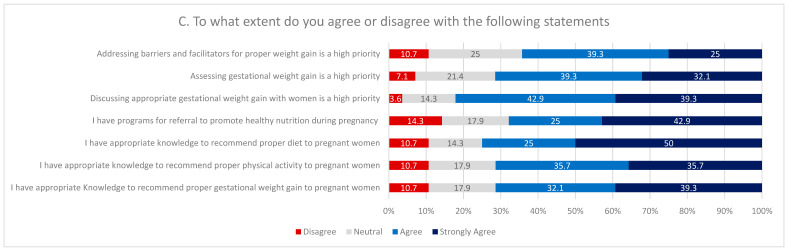
HCPs’ responses to GWG-related practice and perceptions.

Figure 2 summarizes providers’ survey responses on their GWG-related practices and opinions in prenatal care settings. HCPs reported that pregnant women attending antenatal visits seldom ask about recommended GWG (53.6% reported that this never or rarely happens). Similarly, 35.7% of HCPs reported that women rarely or never inquire about how much they should eat, and 42.8% reported that women seldom seek resources on healthy eating during pregnancy.

When asked how often they undertake various activities during GWG counseling in ANC, nearly all HCPs (93%) often or almost always weigh pregnant women at every visit. Additionally, 78.5% frequently discuss vitamin and supplement intake, and 78.6% frequently address appropriate physical activity during pregnancy, often or almost always. However, only 21.4% often or almost always provide personalized GWG targets based on pre-pregnancy BMI. 

HCPs were asked to rate their level of agreement with statements about the perceived priority of GWG counseling items. Most HCPs (82.2%) agreed or strongly agreed that discussing appropriate GWG is a high priority, and 71.4% agreed or strongly agreed that assessing GWG is highly important. Additionally, 64.3% agreed or strongly agreed that addressing barriers and facilitators to proper GWG should be prioritized. Nearly 68.0% reported having established referral programs to dietitians, and over 70% felt confident in their knowledge and resources to provide appropriate recommendations on GWG, diet, and exercise during pregnancy.

## 4. Discussion

In this study, only 25% of women recalled receiving GWG advice from their HCPs, and just 8.8% reported being given target GWG values aligned with the IOM guidelines. This result aligns with HCP reports, among whom only 21% reported routinely providing individualized GWG recommendations during antenatal care visits. Among those who received GWG advice inconsistent with IOM guidelines, underweight and normal-BMI women were more likely to receive advice below the IOM recommendation, whereas overweight and obese women received advice above the IOM recommendations. Only 27.9% of women demonstrated accurate knowledge of the recommended GWG ranges for their BMI. Women who were overweight or obese and those who received accurate advice from HCPs were more likely to identify the appropriate GWG for their BMI category. These findings highlight a significant gap in GWG counseling practices and awareness, indicating limited communication between women and HCPs about proper weight management during pregnancy.

The current results align with previous studies in other settings, which have similarly reported limited GWG counseling and suboptimal awareness among pregnant women and HCPs. Studies from the United States, Canada, and Australia have shown that only 20–40% of pregnant women recall receiving GWG advice consistent with clinical guidelines, and that many healthcare professionals either provide incomplete information or omit counseling altogether [19,20,21]. Other studies [22,23,24] supported these findings and clarified that most clinicians acknowledged the importance of GWG counseling but cited barriers such as time constraints, uncertainty about guidelines, and discomfort discussing mothers’ weight.

In Saudi Arabia, a recent study [25] examined attitudes toward GWG and associated prenatal health behaviors, reporting limited awareness and inconsistent counseling among pregnant women. Similarly, Alweldawi et al. found that more than 70% of pregnant women who participated in the study had poor knowledge about appropriate weight gain during pregnancy and that their knowledge improved significantly following education, underscoring the potential impact of structured counseling. However, the same study demonstrated moderate improvement in women beliefs and attitude towards healthy nutrition and exercise during pregnancy [26].

The demographic characteristics of Saudi pregnant women who maintain a healthy lifestyle during pregnancy include those who are physically active, with low pre-pregnancy BMI, and have high income and educational attainment levels [27].

Recent data from the RAHMA cohort [12] further revealed that nearly half of Saudi women experienced inadequate GWG, highlighting the need for effective provider guidance during antenatal care. Other regional studies, including those from Oman and Gulf countries, have reported comparable trends, with GWG often deviating from recommended ranges, counseling remaining suboptimal, and GWG recognized as a risk factor for pregnancy-related adverse outcomes [28,29].

While antenatal care presents an excellent opportunity for health education, including maternal nutrition and target weight gain, this potential is often not realized due to the demands of busy clinics or the lack of ongoing education for HCP [30,31].

A systematic review of the provision and uptake of antenatal care [32] concluded that effective antenatal care depends on adequate resources and staffing. It requires time to offer flexible, private appointments, along with a compassionate staff who establish effective, culturally appropriate connections with local communities. Furthermore, healthcare professionals need sufficient training and education to perform their duties effectively. Hence, to deliver effective antenatal care in Saudi Arabia, service reform with task shifting to nurses and nutritionists, which has proven effective and safe, is essential to address maternal weight-related clinical problems [33]. Triaging women at the first clinical station where women are weighted using charts based on individual woman BMI, will reduce the load of work by targeting those in need of further management of weight [34], who can be referred to a nurse/midwife clinic for effective individualized discussion of weight management which proved to be effective in many settings [35,36].

Establishment of educational classes for couples who are expecting a baby is an effective approach for safe environment for nutrition health education and discussion of optimum weight gain during pregnancy [37].

In the current study, women with higher BMI were more likely to receive GWG advice and correct recommendations than those with lower BMI. Underweight and normal-weight women mainly received inadequate advice, whereas overweight and obese women more often received advice that exceeded recommendations. These findings align with other studies showing that GWG advice patterns are based on women’s BMI [38]. Additionally, these results may explain the high prevalence of inadequate GWG among Saudi women, especially among underweight and normal-weight individuals, which reached 60%, while excess GWG occurred in 30% of overweight and obese women [12].

Further evidence suggests that both healthcare professionals’ provision of GWG guidance and women’s knowledge of appropriate GWG are significant predictors of achieving guideline-concordant weight gain. Many studies report that women who received IOM-based GWG recommendations from their HCPs were significantly more likely to set a concordant GWG goal than those who did not [24,39]. Furthermore, HCP advice is associated with women’s intention to reach their GWG goal within the IOM guidelines [22]. Another study examining the effect of HCP advice found that not receiving IOM-consistent advice increased the risk of women gaining weight beyond recommended levels [9]. These findings reinforce the importance of effective communication between women and HCPs to ensure understanding and adherence to GWG targets, thereby promoting healthier pregnancy outcomes.

Contrary to these findings, a recent study of 9353 nulliparous women found that advice on appropriate GWG was not predictive of achieving the target weight gain. The study suggested that multifaceted interventions targeting multiple mediators of goal-setting success could help women reach their GWG goals [23].

The sensitivity analysis in this study revealed that the effect of pre-pregnancy BMI was not particularly strong in determining the appropriate GWG for pregnant women; it also showed that incorrect HCP advice can negatively affect pregnant women’s likelihood of defining their target GWG. These findings need to be interpreted cautiously, as the strong association between “receiving correct advice” and “correctly defining GWG” may partly reflect how women remember and interpret prior information rather than a direct effect of counselling itself.

In the current study, most physicians reported that their most common routine was weighing pregnant women at each antenatal care visit, while discussing details, and that the impact of GWG on pregnancy outcomes was rarely discussed. This aligns with other studies examining the GWG content of antenatal care visits. For example, a study in Australia [40] assessing HCPs’ attitudes toward routine weighing of pregnant women during antenatal visits reported that although most staff supported routine weighing, various concerns were raised, including access to resources and staff, the ability to provide appropriate counseling and evidence-based interventions, and the impact of weighing on the therapeutic relationship [40]. Although many clinicians appreciate the importance of appropriate GWG, many feel reluctant to discuss it with pregnant women. This reluctance stems from fears of causing unnecessary anxiety, beliefs that pregnant women cannot control GWG, or perceptions that weight control interventions during pregnancy are ineffective [41,42,43]. However, many studies have recognized the role of HCP advice on GWG and highlighted HCPs as the most trusted source of information on GWG for pregnant women [44].

### 4.1. Implication to Practice and Research

Given the well-established importance of achieving appropriate GWG for both maternal and infant health and for reducing long-term obesity risk, the findings of this study suggest:

1. Given that only 25% of women recalled receiving guidance on GWG from their HCPs, it is crucial to regularly monitor GWG during pregnancy and counsel mothers to achieve appropriate GWG based on their BMI.
2. Saudi mothers should be the center of preconception, antenatal, and postnatal care, and they should be empowered by knowledge to achieve the proper pre-pregnancy weight and adequate GWG to have healthy pregnancy outcomes.
3. Efforts should also aim to align women’s beliefs and expectations about ideal weight gain during pregnancy more closely with evidence-based recommendations.
4. The integration of telemedicine and the utilization of mobile applications to educate mothers on various aspects of antenatal care, including appropriate weight gain during pregnancy, presents a promising opportunity to enhance women’s knowledge and engagement in their prenatal care.
5. Training programs tailored for HCPs to enhance their confidence in discussing GWG are required, and these programs should account for time constraints while providing proper GWG counseling. The development of standardized tools or resources to facilitate GWG counseling and the integration of GWG counseling into routine ANC protocols can improve practice and service quality.
6. ANC providers should engage in continuing professional development to stay informed about the rapidly advancing aspects of maternal care. This includes understanding appropriate GWG guidelines, ensuring adequate maternal nutrition, and providing proper counseling to pregnant women.
7. The study revealed that physicians, rather than nurses or midwives, are primarily responsible for providing advice on GWG. This observation suggests a potential underutilization of other healthcare professionals who could significantly contribute to discussions on maternal nutrition and weight management during pregnancy.
8. Exploring mothers’ and their families’ perspectives on healthy nutrition during pregnancy and on anticipated GWG is crucial. This understanding will establish a robust foundation for effective evidence-based interventions, ensuring that the mother remains at the center of care.
9. Further research should focus on mothers and their families’ perceptions of weight gain during pregnancy, community norms and beliefs about healthy weight, and the effects of mothers’ weight on pregnancy outcomes. Such an investigation will provide essential knowledge to establish an effective counseling program.

### 4.2. Strengths and Limitations

The study addresses a major research gap by examining GWG awareness and counseling practices in Saudi Arabia, where such data have been limited. It explores both women’s perspectives and healthcare provider practices, providing a more holistic understanding of GWG counseling dynamics. Additionally, including women across different BMI categories enhances the generalizability of the results within the studied population. Nevertheless, several limitations should be considered; its cross-sectional design precludes causal inference among counseling practices, women’s knowledge, and GWG outcomes. Reliance on self-reported pre-pregnancy weight and counseling recall may have introduced recall and reporting bias.

Another limitation of the current study is that the healthcare provider sample was small, diverse in professional background, and recruited through purposive sampling. While the providers’ perspective is valuable, the imbalance between the large sample of women and the limited HCP sample makes it difficult to draw system-level conclusions.

## 5. Conclusions

A significant proportion of women report receiving no guidance from HCPs regarding GWG. Among those who do receive advice, the majority indicate that the recommendations were inconsistent with current guidelines. Given that both weight gain targets and practitioner guidance are modifiable, improving these factors offers a critical opportunity to promote healthier pregnancy outcomes.

## Figures and Tables

**Table 1:** Characteristics of the Study Participants.

		N	(%)
Participating Women, N = 1151			
**Age (years)**	(average ± standard deviation)	36.09 ± 9.04	
*Missing*		*12*	
**Education**	School	198	(17.3)
	University	816	(71.3)
	Master/PhD	131	(11.4)
*Missing*	6	*6*	
**Occupation**	Student	91	(7.9)
	Housewife	511	(44.6)
	Employee	545	(47.5)
*Missing*		*4*	
**Family Income**	Enough income	697	(60.6)
	Enough and save	281	(24.59)
	Not enough	167	(14.81)
*Missing*		*6*	
**Marital Status**	Married	1072	(93.10)
	Unmarried	77	(6.90)
*Missing*		*2*	
**Pregnancy Status**	Currently pregnant	177	(15.38)
	Pregnant within last year	970	(84.62)
*Missing*		*4*	
**Pre-pregnancy BMI (kg/m^2^)**	<18.5	41	(3.6)
	18.5–24.9	548	(47.6)
	25–29.9	381	(33.1)
	≥30	181	(15.7)
**HCPs, N = 28**			
**Profession**	General Practice/Family Medicine	9	(32.1)
	Obstetrics and Gynecology	15	(53.6)
	Nurses	3	(10.7)
	Dietician	1	(3.6)
**Level of Care**	Primary Healthcare Centers	21	(75.0)
	Hospitals	7	(25.0)

Data are presented as frequencies (N) and percentages (%). BMI: Body Mass Index.

**Table 2:** Seeking medical advice about Gestational weight gain among the participating women, categorized by pre-pregnancy BMI.

		Total N = 1151		Pre-Pregnancy Body Mass Index (BMI)								*p*-Value
				<18.5 kg/m^2^ N = 41		18.5–24.9 kg/m^2^ N = 548		25–29.9 kg/m^2^ N = 381		≥30 kg/m^2^ N = 181		
		N	%	N	%	N	%	N	%	N	%	
GWG target as advised by healthcare provider	Non-Specific	850	74.5	29	70.7	416	76.2	270	72.4	135	74.6	<0.01 #
	12.5–18 kg	26	2.3	1	2.4	20	3.7	3	0.8	2	1.1	
	11.5–16 kg	55	4.8	2	4.9	29	5.3	21	5.6	3	1.7	
	7–11.5 kg	102	8.9	5	12.2	41	7.5	43	11.5	13	7.2	
	5–9 kg	108	9.5	4	9.8	40	7.3	36	9.7	28	15.5	
	*Missing*	*10*										
Physician’s advice consistent with IOM	Yes	101	8.8	1	2.4	29	5.3	43	11.5	28	15.5	<0.01 **¶**
Seeking information about GWG during pregnancy	Yes	624	54.5	25	61.0	289	52.8	205	54.7	105	58.0	0.53 **¶**
	No	520	45.5	16	39.0	258	47.2	170	45.3	76	42.0	
	*Missing*	*7*										
Dr. advised me to follow certain diet	Yes	272	23.8	6	14.6	108	19.8	102	27.2	56	30.9	<0.01 **¶**
	No	871	76.2	35	85.4	438	80.2	273	72.8	125	69.1	
	*Missing*	*8*										
Dr. advised me to contact dietitian	Yes	183	16.0	8	19.5	84	15.4	60	16.0	31	17.2	0.86 **¶**
	No	960	84.0	33	80.5	463	84.6	315	84.0	149	82.8	
	*Missing*	*8*										
Correct expectation of GWG	Incorrect	810	72.1	37	90.2	461	84.3	230	64.8	82	45.3	<0.01 **¶**
	Correct	314	27.9	4	9.8	86	15.7	125	35.2	99	54.7	
	*Missing*	*27*										

Data are displayed as frequencies and percentages. GWG: Gestational Weight Gain; IOM: Institute of Medicine. ¶: chi-square test used; #: Fisher’s exact test used.

**Table 3:** Factors affecting the definition of the correct GWG by participating women.

	Crude Odds Ratio (95%C.I.)	Adjusted Odds Ratio (95% C.I.) Total Population (N = 1095)	Adjusted Odds Ratio (95% C.I.) Pregnant Women (N = 171)
**Age (in years)**	1.01 (0.99–1.09)	1.02 (0.98–1.022)	0.98 (0.90–1.06)
**Education**			
Postgraduate	1	1	1
University	1.24 (0.81–1.91)	1.31 (0.84–2.03)	0.84 (0.18–3.90)
School	1.14 (0.69–1.90	0.82 (0.43–1.56)	2.24 (0.31–16.20)
**Occupation**			
Employee	1	1	
Student	0.55 (0.31–0.96) *	1.03 (0.54–1.97)	7.92 (0.86–73.03)
Housewife	0.90 (0.68–1.17)	1.25 (0.65–2.40)	10.16 (0.99–99.7)
**Income**			
Enough and save	1.10 (0.72–1.66)	1.45 (1.01–2.07) *	1.44 (1.01–2.07) *
enough	0.81 (0.55–1.17)	0.96 (0.63–1.48)	0.20 (0.60–1.01)
Not enough	1	1	1
**Pre-pregnancy BMI**			
18.6–24.9 kg/m^2^	1	1	1
25–29.9 kg/m^2^	2.91 (2.12–4.00) *	2.84 (2.01–4.03) *	1.91 (0.73–5.09)
30+ kg/m^2^	6.47 (4.46–9.93) *	5.85 (3.88–8.82) *	2.29 (0.79–6.72)
<18.5 kg/m^2^	0.58 (0.20–1.66)	0.70 (0.24–1.11)	0
**Pregnancy status**			
Currently pregnant	1	1	-
Within last year	0.99 (0.96–1.45)	0.74 (0.47–1.16)	-
**Sought advice**			
Yes	1	1	1
No	0.72 (0.55–0.94) *	0.77 (0.58–1.04)	0.24 (0.09–0.61) *
**Received medical advice**			
Correct advice	7.50 (4.66–12.07) *	7.13 (4.35–11.66) *	3.36 (0.74–15.66)
Incorrect advice	0.39 (0.25–0.62) *	0.34 (0.21–0.56) *	0.05 (0.01–0.50) *
Non-specific	1	1	1
**Model’s Summary**		Cox& Snell R square = 0.18, Nagelkerke R Square 0.26	Cox& Snell R square = 0.24, Nagelkerke R Square 0.34

BMI: Body Mass Index, C.I.: Confidence Interval. * *p*-value < 0.05.

## Data Availability

Data from this study is available to researchers upon request and approval of Institutional Review Board at Princess Nourah bint Abdulrahaman University (irb@pnu.edu.sa). The request and approval of data sharing are independent from the research team.

## Supplementary Materials

The following supporting information can be downloaded at: https://www.mdpi.com/article/10.3390/healthcare14030403/s1, Supplementary File S1: The Arabic and English version of the questionnaire is available.
