# Supplementary material for: Gestational Weight Gain Counseling Insights from Healthcare Providers and Saudi Women: Riyadh Mother and Baby Follow-Up Study (RAHMA Explore)
Source: Healthcare (Basel). 2026 Feb 5;14(3):403. doi: 10.3390/healthcare14030403 (PMC12897361; doi:10.3390/healthcare14030403)
Supplement: Supplementary file 1 [file healthcare-14-00403-s001.zip › healthcare-4097727-supplementary.pdf]

Gestational Weight Gain questionnaire

العمر
Age

مستوى التعليم<br>**Education**	تعليم مدرسي<br>**School**	تعليم جامعي<br>**University**	تعليم ما بعد الجامعي (ماجستير /دكتوراة<br>**Higher education (Master or PhD)**
الوظيفة<br>**Occupation**	موظفة<br>**Employee**	ربة منزل<br>**Housewife**	طالبة<br>**Student**
مستوى دخل الاسرة<br>**Family Income**	لا يكفي<br>**Not Enough**	يكفي وندخر<br>**Enough and save**	كافي<br>**Enough**
الحالة الاجتماعية<br>**Marital Status**	أرملة أو مطلقة<br>**Widowed/Divorced**	متزوجة<br>**Married**	

متى كان آخر حمل لك؟
When was your latest pregnancy? — خلال العام الماضي **Within the last year** — حامل حاليا **Currently pregnant**

ما هو وزنك قبل الحمل الحالي /قبل آخر حمل؟
What was your weight before the latest pregnancy?

ما هو طولك؟
What is your height?

ما هي الزيادة المثالية في الوزن اثناء الحمل لسيدة في مثل طولك ووزنك؟
What is the optimal gestational weight gain for a woman of your weight and height?

٥-٩ كجم<br>**5-9 kg**	كجم ٧-١١.٥<br>**7-11.5 kg**	كجم ١١.٥-١٦<br>**11.5-16 kg**	كجم ١٢.٥-١٨<br>**12.5 -18 kg**

هل قدم لك طبيبك مقترحا أو هدفا محددا لمقدار زيادة الوزن أثناء الحمل؟
Has your doctor advised you with a certain target of gestational weight gain for you? — نعم **Yes** — لا **No**

ما هو الوزن الذي نصحك به الطبيب للوصول إليه بنهاية الحمل؟
What weight range did the healthcare provider advise you to reach?

لم ينصحني الطبيب بزيادة وزن معينة<br>Doctor did not give me certain advice	٥-٩ كجم<br>5-9 kg	كجم ٧-١١.٥<br>7-11.5 kg	كجم ١١.٥-١٦<br>11.5-16 kg	كجم ١٢.٥-١٨<br>12.5 -18 kg

**هل بحثت عن معلومات (بما في ذلك على الإنترنت) أو سألت أي شخص عن مقدار الوزن الذي يجب أن تكتسبيه أثناء الحمل؟**<br>**During this pregnancy, have you looked for information (including on the internet) or asked anyone about how much weight you should gain during pregnancy?**	☐عم<br>Yes	لا<br>No
**هل نصحك الطبيب باتباع حمية معينة اثناء الحمل ؟**<br>**Has your healthcare provider advised you to follow certain diet plan?**	☐عم<br>Yes	لا<br>No
**هل نصحك الطبيب بالمتابعة مع متخصص في التغذية؟**<br>**Has your healthcare provider referred you to a dietitian?**	☐عم<br>Yes	لا<br>No
